# miR-155 T/A (rs767649) and miR-146a A/G (rs57095329) single nucleotide polymorphisms as risk factors for chronic hepatitis B virus infection among Egyptian patients

**DOI:** 10.1371/journal.pone.0256724

**Published:** 2021-08-26

**Authors:** Enas M. Hefzy, Noha A. Hassuna, Olfat G. Shaker, Mohamed Masoud, Tebyan A. Abelhameed, Tarek I. Ahmed, Nada F. Hemeda, Mohammed A. Abdelhakeem, Rania H. Mahmoud

**Affiliations:** 1 Faculty of Medicine, Department of Medical Microbiology and Immunology, Fayoum University, Fayoum, Egypt; 2 Faculty of Medicine, Department of Medical Microbiology and Immunology, Minia University, Minia, Egypt; 3 Faculty of Medicine, Department of Medical Biochemistry and Molecular Biology, Cairo University, Cairo, Egypt; 4 Faculty of Medicine, Department of Public Health, Fayoum University, Fayoum, Egypt; 5 Department of Biotechnology, Africa City of Technology, Khartoum, Sudan; 6 Faculty of Medicine, Department of Internal Medicine, Fayoum University, Fayoum, Egypt; 7 Faculty of Agriculture, Department of Genetics, Fayoum University, Fayoum, Egypt; 8 Faculty of Medicine, Department of Clinical Pathology, Minia University, Minia, Egypt; 9 Department of Medical Biochemistry and Molecular Biology, Fayoum University, Fayoum, Egypt; Centre de Recherche en Cancerologie de Lyon, FRANCE

## Abstract

Genetic variants in microRNAs (miRNAs) can alter the miRNAs expression and/or function, accordingly, affecting the related biological pathways and disease risk. Dysregulation of miR-155 and miR-146a expression levels has been well-described in viral hepatitis B (HBV). In the current study, we aimed to assess rs767649 T/A and rs57095329 A/G polymorphisms in miR-155, and miR-146a genes, respectively, as risk factors for Chronic HBV (CHBV) in the Egyptian population. Also, we aimed to do in silico analysis to investigate the molecules that primarily target these miRNAs. One hundred patients diagnosed as CHBV and one hundred age and sex-matched controls with evidence of past HBV infection were genotyped for miR-155 (rs767649) and miR-146a (rs57095329) using real-time polymerase chain reaction. The rs767649 AT and AA genotypes in CHBV patients confer four folds and ten folds risk respectively, as compared to control subjects [(AOR = 4.245 (95%CI 2.009–8.970), *p*<0.0001) and AOR = 10.583 (95%CI 4.012–27.919), *p*<0.0001, respectively)]. The rs767649 A allele was associated with an increased risk of developing CHBV (AOR = 2.777 (95%CI 1.847–4.175), *p*<0.0001). There was a significant difference in the frequency of rs57095329 AG and GG genotypes in CHBV patients compared to controls. AG and GG genotypes showed an increase in the risk of developing CHBV by about three and six folds respectively [AOR = 2.610 (95%CI 1.362–5.000), *p* = 0.004] and [AOR = 5.604 (95%CI 2.157–14.563), *p*<0.0001].We concluded that rs57095329 and rs767649 SNPs can act as potential risk factors for the development of CHBV in the Egyptian population.

## Introduction

Infection by the hepatitis B virus (HBV) is one of the most common viral infections worldwide especially in developing countries [1]. In Egypt, HBV prevalence was 1.4% among ages ranged 15–59 in Egypt Health Issues Survey, 2015 [2]. In another survey in Southern Upper Egypt, about 4.4% of the studied population had chronic HBV(CHBV) [3]. In adults, less than 5% of infected healthy persons will develop chronic infections; and 20–30% of chronically infected adults will develop fibrosis, cirrhosis, and/or liver cancer [4]. Many factors can contribute to the progression of CHBV infection.

Genetics is considered as one of the most important factors influencing the clinical course of HBV infection. MicroRNAs (miR) are small non-coding ribonucleic acids formed nearly from 21–25 nucleotides that regulate the expression of protein-coding genes post-transcriptionally by affecting the translation and degradation of target mRNAs [5]. They regulate the host-virus interaction through suppression of viral infection pathways [6]. MiRNAs have a crucial role in the pathogenesis of various hepatic diseases, including chronic viral hepatitis [7].

Genetic variation in the regulatory regions in miRNAs could change their expression levels and this leads to phenotypic diversity [8]. Single nucleotide polymorphisms (SNPs) are variations in a single nucleotide that happen at a particular site in the DNA. SNPs in genes coding miRNAs (miR SNPs) have been found to affect the expression, processing, and functions of these miRNAs [9]. A strong association between these SNPs and disease progression, diagnosis, prognosis, and drug response has been reported [10].

The miR-155 was first found within the B-cell integration cluster non-coding RNA on chromosome 21 in the human genome [11]. It was reported that miR-155 plays an immense role in the regulation of host antiviral immunity and the development of HBV-associated liver diseases. It regulates the production of interferon against HBV via the JAK-STAT signaling and thus participates in innate antiviral immune responses [12]. The rs767649 is a functional variant found in the flanking region of the miR-155 gene that may lead to a poor prognosis and an increased risk of hepatocellular carcinoma [5].

The miR-146a gene resides on chromosome 5q33.3 [13]. The promoter of the miR-146a encoding gene has several binding sites for the nuclear factor- alpha B (NF-αB) transcription factor that plays a significant role in the immune response [14,15]. Unsurprisingly, numerous studies have implicated their association with the pathogenesis of autoimmune diseases [7]. Within the miR-146a gene, rs57095329 A/G in the promoter region is thought to reduce the expression levels of miR-146a [8]. Interestingly, miR-146a A/G SNP (rs57095329) has been associated with reducing the attachment to E26 transforming sequence 1 (ETS-1) transcription factor and affected the risk of systemic lupus erythematosus [9].

Dysregulation of the miR-146a and miR-155 expression levels has been well-described in viral hepatitis B [16]. However, it has not been reported yet, up to our knowledge, whether there is an association between the pathogenesis of CHBV infection and the miR-146a and miR-155 gene SNPs at loci rs57095329 A/G and rs767649 A/T respectively. We hypothesized that these potentially functional genetic variants may be associated with the progression of CHBV in the Egyptian population.

The current study aimed to assess these SNPs as risk factors for CHBV in the Egyptian population, as well as their relationship with the patients’ clinical characteristics. Also, we aimed to do in silico analysis to explore the molecules that primarily targeted these miRNAs, and if rs57095329 A/G and rs767649 A/T may contribute to alterations in cellular function that ultimately lead to disease.

## Results

### Basic and clinical characteristics of study subjects

**Table 1** shows the basic and clinical characteristics of the study groups. There was no statistically significant difference between cases and control as regards age (39.6±13 vs. 38.8±11.8, *p* = 0.661) and sex (*p* = 0.744).

**Table 1:** Basic and clinical characteristics of study subjects.

	Cases (N = 100)	Controls (N = 100)		P-value
	Mean ± SD			
**Age (years)**	39.6±13	38.8±11.8		0.661
**BMI (kg/m** ^**2**^ **)**	27.6±3.9	28.8±2.1		0.260
**Hb(g/dl)**	13.5±1.9	13.2±1.2		0.688
**TLC (/mm** ^3^ **)**	6442.8±2006.5	8500±1500.3		<0.0001*
**PLT (/mm** ^3^ **)**	220020±98271	342000±1250		<0.0001*
**Creatinine (mg/dl)**	0.9±0.2	0.8±0.3		0.077
**ALT(IU/L)**	46.1±9.9	39.3±7.5		<0.0001*
**AST(IU/L)**	41.2±7.2	32.7±6.4		<0.0001*
**Total Bilirubin (mg/dl)**	0.7±0.3	0.75±0.21		<0.0001*
**Albumin (g/dl)**	4.2±0.6	4.6±0.45		0.006*
**INR**	1.1±0.3	0.8±0.2		<0.0001*
	**N(%)**			
**Gender**				
Female (N%)	24(24.0)	26(26.0)		0.744
Male	76(76.0)	74(74.0)		
**Viral load**				
<10 IU/mL	36(36.0)			
≥10 IU/mL	64(64.0)			
**Fibroscan(Transient elastography**)				
F0	42(42.0)			
F1-F2	42(42.0)			
F3-F4	16(16.0)			

*Statistical significance was considered at *p*-value ≤0.05; SD, standard deviation; BMI, body mass index; Hb, hemoglobin; TLC, total leukocyte count; PLT, Platelets; ALT, alanine aminotransferase; AST, aspartate aminotransferase; INR, International Normalized Ratio; F0, No liver scarring; F1, Mild liver scarring; F2, Moderate liver scarring; F3, Severe liver scarring; F4, Advanced liver scarring.

### Analysis of the frequency and association between miR-155 T/A polymorphism (rs767649) and miR-146a A/G (rs57095329) and susceptibility to CHBV viral infection

There was a statistically significant difference between the study groups regarding rs767649 genotype distribution as denoted by the Cochran-Armitage test for trend, *p*<0.0001. When compared to TT genotype (the normal genotype), there was about four folds risk of being CHBV when the patient has the mutant AT genotype; [AOR = 4.245 (95%CI 2.009–8.970), *p*<0.0001]. Moreover, there was about ten folds risk of being CHBV when the patient has the mutant AA genotype; [AOR = 10.583 (95%CI 4.012–27.919), *p*<0.0001]. The frequency of the AT and AA genotypes was higher in CHBV patients than in control (58% vs. 48%) and (30% vs. 10%), respectively **(Table 2**).

**Table 2:** Analysis of the frequency and association between miR-155 T/A polymorphism (rs767649) and miR-146a A/G (rs57095329) and susceptibility to chronic hepatitis B viral infection.

	Cases (N = 100)	Controls (N = 100)	Adjusted OR, (95% CI), p-value
	N (%)		
**rs767649**			
TT	12(12.0%)	42(42.0%)	
AT	58(58.0%0	48 (48.0%)	4.245 (2.009–8.970), <0.0001*
AA	30(30.0%)	10(10.0%)	10.583 (4.012–27.919), <0.0001*
**Dominant**			
TT	12(12.0%)	42(42.0%)	
AT+AA	88(88.0%)	58(58.0%)	5.297 (2.567–10.929), <0.0001*
**Recessive**			
TT+AT	70(70.0%)	90(90.0%)	
AA	30(30.0%)	10(10.0%)	3.841 (1.748–8.438), 0.001*
**Allele**			
T	82(41.0%)	132(66.0%)	
A	118(59.0%)	68(34.0%)	2.777 (1.847–4.175), <0.0001*
**rs57095329**			
AA	21(21.0%)	45(45.0%)	
AG	56 (56.0%)	46(46.0%)	2.610 (1.362–5.000), 0.004*
GG	23(23.0%)	9(9.0%)	5.604 (2.157–14.563), <0.0001*
**Dominant**			
AA	21(21.0%)	45(45.0%)	
AG+GG	79(79.0%)	55(55.0%)	3.061 (1.638–5.721), <0.0001*
**Recessive**			
AA+AG	77(77.0%)	91(91.0%)	
GG	23(23.0%)	9(9.0%)	3.078 (1.311–7.231), 0.010*
**Allele**			
A	98(49/0%)	136(68.0%)	
G	102(51.0%)	64(32.0%)	2.195 (1.456–3.308), <0.0001*

*Statistical significance was considered at *p* value ≤0.05; OR, Odds Ratio; CI, Confidence interval; HWE in control: **rs767649;** chi-squared value = 0.483, *p*-value = 0.487; **rs57095329;** chi-squared value = 0.325, *p*-value = 0.569.

In the dominant model, there was about five folds risk of being CHBV when the patient had the mutant TT or AT genotypes, the AOR was 5.297 (95%CI 2.567–10.929), *p*< 0.0001. The frequency of the TT or AT genotypes was higher in CHBV than in control (88% vs. 58%) **(Table 2**).

In the recessive model participants with AA genotype had a higher risk, about three times, for being CHBV, the AOR was 3.841 (95% CI 1.748–8.438), (*p* = 0.001). The frequency of AA was higher in CHBV than in control (30% vs. 10%) **(Table 2**).

Patients with CHBV had a higher frequency of allele A than control (59% vs. 34%). The risk of CHBV increased by about two and a half [AOR = 2.777 (95%CI 1.847–4.175), *p*<0.0001] when the patients had allele A **(Table 2**).

As regards rs57095329, the genotypes distribution had a statistically significant difference between cases and control as denoted by the Cochran-Armitage test for trend, *p*<0.0001. As compared to the normal AA genotype, there was about two and a half folds risk of being CHBV when the patient has the AG genotype; [AOR = 2.610 (95%CI 1.362–5.000), *p* = 0.004]. Also, there was about five folds risk of being CHBV when the patient has GG genotype; [AOR = 5.604 (95%CI 2.157–14.563, *p*<0.0001). The occurrence of AG and GG was higher in CHBV patients than in control (56% vs. 46%) and (23% vs. 9%), respectively **(Table 2**).

In the dominant model, there was about three folds risk of having CHBV when the patient was GG or AG as AOR was 3.061 (95%CI 1.638–5.721), (*p*<0.0001). The frequency of GG or AG was higher in CHBV (78%) than in control (55%) **(Table 2**).

In the recessive model, participants with GG genotype had a higher risk, about three times, for being CHBV. The AOR was 3.078 (95%CI 1.311–7.231), (*p* = 0.01). The frequency of GG was higher in CHBV than in control (22% vs. 9%) **(Table 2**).

Patients with CHBV had a higher level of allele G than control (51% vs. 32%). The risk of CHBV increased by about two folds [AOR = 2.195 (95%CI 1.456–3.309, *p*<0.0001] when the patients had allele G **(Table 2**).

Both polymorphisms were not deviated from HWE in control (*p*-value = 0.487 and *p*-value = 0.569 for rs767649 and rs57095329, respectively) **(Table 2**).

### The miR-155 T/A polymorphism (rs767649) and the characteristics of cases with CHBV infection

As regards the relation between the miR-155 T/A polymorphism (rs767649) and the characteristics of cases with CHBV infection, **Table 3** shows that there were statistically significant differences between genotypes TT, AT, and AA regarding alanine aminotransferase (ALT) (39.7±13.4, 46.6±8.3 vs. 47.7±10.4 IU/L respectively) (*p* = 0.048). There were no statistically significant differences between genotypes as regards other study parameters (p>0.05).

**Table 3:** The miR-155 T/A polymorphism (rs767649) to the characteristics of cases with chronic hepatitis B viral infection.

	Genotype						P-value	Alleles				P-value
	TT (N = 12)		AT (N = 58)		AA (N = 30)			T (N = 82)		A (N = 118)		
	Mean±SD							Mean±SD				
**BMI (kg/m** ^**2**^ **)**	27.6±3.8		27.4±4.2		28.1±3.4		0.672	27.4±4.1		27.8±3.8		0.551
**Hb(g/dl)**	13.5±0.8		13±1.9		13.9±2.1		0.102	13.5±1.8		13.5±2		0.976
**TLC (/mm** ^3^ **)**	5966.7±1285.8		6495.2±2175.5		6532±1925.3		0.683	6340.5±1959.1		6513.9±2035.6		0.548
**PLT (/mm** ^3^ **)**	189333.3±88046.8		223896.6±91199.2		224800±115047.9		0.519	213780.5±90602.5		224355.9±103022.7		0.454
**Creatinine (mg/dl)**	0.8±0.2		0.8±0.2		0.9±0.3		0.051	0.8±0.2		0.84±0.2		0.166
**ALT(IU/L)**	39.7±13.4		46.6±8.3		47.7±10.4		**0.048** *	44.5±10.4		47.2±9.4		0.065
**AST(IU/L)**	38.8±14.1		40.8±5.9		42.9±5.3		0.202	40.2±8.9		41.9±5.7		0.108
**Total Bilirubin (mg/dl)**	0.7±0.3		0.7±0.2		0.6±0.3		0.813	0.69±0.26		0.67±0.28		0.639
**Albumin (g/dl)**	4.2±0.3		4.3±0.5		4±0.9		0.052	4.3±0.4		4.1±0.8		0.105
**INR**	1±0		1.1±0.1		1.2±0.5		0.150	1.1±0.1		1.1±0.3		0.103
	**N (%)**							**N (%)**				
**Gender**												
Female	0(0.00%)		16(27.60%)		8(26.70%)		0.116	16(19.5%)		32(27.1%)		
Male	12(100.00%)		42(72.40%)		22(73.30%)			66(80.5%)		86(72.9%)		
**Viral load**												
<10 IU/mL	2	(16.7%)	22	37.9%	12	40.0%	0.325	26	31.7%	46	39.0%	0.292
≥10 IU/mL	10 (83.3%)		36 (62.1%)		18 (60.0%)			56(68.3%)		72(61.0%)		
**Fibroscan(Transient elastography**)												
F0	4(33.30%)		24(41.40%)		14(46.70%)		0.294	32(39.0%)		52(44.1%)		0.215
F1-F2	8(66.70%)		24(41.40%)		10(33.30%0			40(48.8%)		44(37.3%)		
F3-F4	0(0.00%)		10(17.20%)		6(20.00%)			10(12.2%)		22(18.6%)		

*Statistical significance was considered at *p*-value ≤0.05; BMI, body mass index; Hb, hemoglobin; TLC, total leukocyte count; PLT, Platelets; ALT, alanine aminotransferase; AST, aspartate aminotransferase; INR, International Normalized Ratio; F0, No liver scarring; F1, Mild liver scarring; F2, Moderate liver scarring; F3, Severe liver scarring; F4, Advanced liver scarring (cirrhosis).

### The miR-146a A/G polymorphism (rs57095329) and the characteristics of cases with CHBV infection

For the miR-146a A/G polymorphism (rs57095329) relation to the characteristics of cases with CHBV infection, there was a statistically significant relationship between sex and genotypes, *p* = 0.032. Regarding Hb, there was a statistically significant difference between the three genotypes; AA, AG, and GG (13.3±1.4, 13.1±2.0 vs. 14.6±1.8 g/dl respectively, *p* = 0.005).

Also, total bilirubin was significantly different between genotypes (0.6±0.3, 0.7±0.3 vs. 0.6±0.2 mg/dl respectively, *p* = 0.034). In contrast, there were no statistically significant differences between genotypes as regards other study parameters (*p*>0.05). Likewise, allele G had a higher level of Hb than allele A (13.7±2 vs. 13.2±1.7 g/dl respectively, *p* = 0.032) **(Table 4**).

**Table 4:** The miR-146a A/G polymorphism (rs57095329) to the characteristics of cases with chronic hepatitis B viral infection.

	Genotype				Alleles		
	AA (N = 21)	AG (N = 56)	GG (N = 23)	P-value	A (N = 98)	G (N = 102)	P-value
	Mean±SD				Mean±SD		
**Age**	37.0±11.5	38.9±12	44.8±15.7	0.174	38±11.7	41.1±13.8	0.094
**BMI (kg/m** ^**2**^ **)**	26.9±4.2	27.5±3.9	28.5±3.5	0.433	27.3±4	27.9±3.7	0.232
**Hb (g/dl)**	13.3±1.4	13.1±2	14.6±1.8	**0.005** *	13.2±1.7	13.7±2	**0.032** *
**TLC (/mm** ^3^ **)**	6500.0±1956.0	6171.4±2130.3	7051.3±1647.9	0.208	6312.2±2043.9	6568.2±1961.6	0.367
**PLT (/mm** ^3^ **)**	215285.7±107695.0	208892.9±90745.3	251434.8±104682.6	0.212	211632.7±97273.1	228078.4±98543.2	0.237
**Creatinine (mg/dl)**	0.8±0.1	0.9±0.3	0.9±0.1	0.186	0.8±0.2	0.9±0.2	0.137
**ALT(IU/L)**	45.0±7.9	47.4±9	44.0±13.1	0.329	46.3±8.5	45.8±11	0.709
**AST(IU/L)**	40.6±5.2	41.5±5.8	41.0±11.1	0.884	41.1±5.6	41.3±8.5	0.882
**Total Bilirubin (mg/dl)**	0.6±0.3	0.7±0.3	0.6±0.2	**0.034** *	0.66±0.29	0.69±0.25	0.319
**Albumin (g/dl)**	4.4±0.3	4.1±0.8	4.3±0.3	0.087	4.2±0.7	4.2±0.6	0.760
**INR**	1.2±0.5	1.1±0.1	1±0.1	0.096	1.1±0.4	1.1±0.1	0.053
	**N (%)**				**N (%)**		
**Gender**							
Female	5(23.80)	18(32.10)	1(4.30)	**0.032** *	28(28.60)	20(19.60)	0.138
Male	16(76.20)	38(67.90)	22(95.70)		70(71.40)	82(80.40)	
**Viral Load**							
<10 IU/mL	7(33.3)	22(39.3)	7(30.4)	0.727	36(36.7)	36(35.3)	0.832
≥10 IU/mL	14(66.7)	34(60.7)	16(69.6)		62(63.3	66(64.7)	
**Fibroscan(Transient elastography**)							
F0	5(23.8)	26(46.4)	11(47.8)	0.066	36(36.7)	48(47.1)	0.302
F1-F2	14(66.7)	18(32.1)	10(42.5)		46(46.9)	38(37.3)	
F3-F4	2(9.5)	12(24.1)	2(8.7)		16(16.3)	16(15.7)	

*Statistical significance was considered at *p*-value ≤0.05; BMI, body mass index; Hb, hemoglobin; TLC, total leukocyte count; PLT, Platelets; ALT, alanine aminotransferase; AST, aspartate aminotransferase; INR, International Normalized Ratio; F0, No liver scarring; F1, Mild liver scarring; F2, Moderate liver scarring; F3, Severe liver scarring; F4, Advanced liver scarring (cirrhosis).

### In silico analysis of the effect of rs767649 SNPs on miR-155 gene and miR-146a gene rs57095329 SNPs on the transcription factors (TFs) binding sites

All data relating to the miR-155 rs767649 SNP and the miR-146a rs57095329 SNP were gathered from dbSNP in the National Center of Biotechnology Information database (https://www.ncbi.nlm.nih.gov/snp/). The miR-155 is located on chromosome 21 in humans. The miR-155 rs767649 SNP located in the promoter region of the miR-155 gene in which the T allele is replaced by A allele, while the miR-146a located in chromosome 5, and the miR-146a rs57095329 SNP located in the promoter region of miR-146a gene in which A allele replaced by G allele. Accordingly, both SNPs were analyzed using Regulome DB, Haploreg, and Alibaba software.

According to Regulome DB software, the rs767649 can affect the binding sites of IRF1 (the interferon regulatory factor); IRF2, and PRDM1 (PR domain zinc finger protein 1), while rs57095329 SNP could affect the binding sites ELF3 (E74 Like ETS Transcription Factor 3). The Haploreg software showed that the rs767649 affects the binding sites of IRF, MRG1 (melanocyte-specific gene-related gene 1), HOXA9 (Homeobox A9), and PRDM1 and the rs57095329 SNP affected the binding sites of ETS (E26 transforming sequence), PAX-5 (a member of the paired box (PAX) family of transcription factors), and STAT (signal transducer and activator of transcription).

On other hand, Haploreg predicted the reference and altered sequences (S1 Table) which were used in Alibaba software as FASTA format showing the effect of both SNPs on some TFs binding sites. According to Alibaba software, the GCN4 (General control protein) binding site was affected with A allele (mutant allele) and was not affected with the T allele (normal allele) of rs767649 SNP. However, MyoD (myoblast determination protein 1) and ETS-1 binding site was altered in case of G allele (mutant allele) of rs57095329 SNP. In general, it was concluded that the rs767649 SNP affected mainly the IRF and PRDM1 TFs binding sites and the rs57095329 affected the binding sites of the ETS family of TFs. The predicted transcription factors binding sites affected by miR-155 rs767649 and miR-146a rs57095329 using Regulome DB, Haploreg and Alibaba software are represented in S2 Table.

## Discussion

One of the main concerns of the CHBV research groups is to define the molecular factors involved in its development. Several studies have documented that viral infections alter the profiles of host miRNAs, which can influence virus-host interfaces and contributes to viral pathogenesis [17].

Presently, the available data suggest that miRNAs are implicated in both the HBV life cycle and the development of HBV-associated liver diseases [18].

It has been outlined that miR-155 is a transactivational target of nuclear factor kappa B (NF-κB), the NF-κBupregulates miR-155. The upregulated miR-155, through its target inhibitor of nuclear factor kappa-B kinase subunits beta and epsilon (IKKβ and IKKε), leads to the limitation of NF-κB [19]. The NF-κB is a key regulator that shares in "the inflammation–fibrosis–cancer process", and the NF-κB signaling pathway seems to have an essential effect on liver homeostasis, pathophysiology, and regulation of the inflammation–fibrosis–cancer axis [20].

Typically, miR-155 is implicated in defensive immunity when adequately regulated and acts within a range of active immune cell types like T cells, B cells, and natural killer cells [21]. In the current study, we found a statistically significant difference between study groups in the miR-155 T/A (rs767649) SNP, as the patients with the homozygous AA genotype were at 10 fold risk of progression to CHBV and those with heterozygous AT genotype showed a four-fold risk of CHBV development with the A allele being a significant risk factor. Since miR-155 expression levels are lower with the AA genotype [5], our findings are consistent with Sarkar et al and Ge et al, who found that miR-155 is down-regulated in peripheral mononuclear cells in the blood of patients with CHBV infection [22,23].

Generally, several reports have shown that the expression level of miR-146a is influenced by two functional SNPs, rs57095329 A/G, and rs2910164 G/C in the gene encoding miR-146a, resulting in a genetic tendency to different diseases [9,24,25]. The association between the levels of miR-146a and liver disorders was previously stated. MiR-146a up-regulation may lead to dysfunction of HBV-specific T cells with subsequent viral persistence [26]. Furthermore, miR-146a levels were elevated in HBV-loaded Huh-7 hepatocytes (used as a *hepatocyte* culture model), as well as in both mice and patients infected with HBV [27].

In the current work, both the mutant GG and AG genotypes of rs57095329 in miR-146a were associated with a significantly increased risk of developing CHBV than the normal AA genotype. It is known that expression levels of miR-146a with the rs57095329 increase in AA patients compared to GG/GA patients [28]. Our findings are thus consistent with Wen et al [29], who found that miR-146a levels are lower in CHBV compared to acute or chronic liver failure and that miR-146a levels are proportionally related to the severity of liver inflammation. This is due to the immune-modulating effect induced by miR-146a on both innate and adaptive immune responses by negative feedback loops inducing down-regulation of their gene products [30].

The effectors of miR-146a are highly variable depending on the cell type as well as different disease conditions including tumor necrosis factor receptor-associated factor 6, complement factor H, and different toll-like receptor signaling pathways [14,27,31].

Since miR-155 and miR-146a activate several essential signaling proteins as well as transcription factors that control different immune processes and differentiation, it is not shocking that they have a pivotal role during immune responses to HBV infection.

The interaction between TFs and miRNAs in a regulatory network has not been discovered yet. In this study we aimed to do in silico analysis to investigate the molecules that primarily target these miRNAs, and if rs767649 T/A and rs57095329 A/G polymorphisms may contribute to alterations in the cellular function that ultimately lead to disease. Generally, we found that the rs767649 SNP affected mainly its interaction with IRF and PRDM1 transcription factors, and the rs57095329 affected its interaction with the ETS family of TFs.

The investigation of the interaction between these miRNAs and the retrieved TFs is not in the scope of this paper because it is not easy to revise the regulatory mechanisms of both TFs and miRNAs even at the data level, as it is too difficult to accomplish a network involving genes, TFs and miRNAs [32]. Also, these predictable networks may not completely expose the multifaceted gene-regulatory mechanisms, such as, the mode in which a TF indirectly controls a gene through a miRNA. However, the bioinformatics findings of this study can highlight the importance of their study in CHBV patients which is a challenge as the combined regulation of miRNAs and TFs in these cases is expected to be complicated, given that they include not only the relations between these regulatory elements and their target genes but also the interactions between the regulatory elements themselves [32].

To the best of the authors’ knowledge, the present research is the first to note a positive relationship between miR-155 rs767649 and miR-146a rs57095329 SNPs and risk of CHBV and it is the first study done on Egyptian patients as well.

There were some limitations to the current analysis. There was unavoidable selection bias because of the hospital-based as well as the case-control design of the research. Also, there was a lack of evidence on other genetic and environmental risk factors that would lead to the development of CHBV in those individuals, and gene-gene and gene-environment associations could not be analyzed.

## Conclusion

SNPs in miR-155 rs767649 and miR-146a rs57095329 are associated with the risk of development of CHBV in Egyptian patients. Such novel results will be of clinical significance and could illuminate the role of miR-155 and miR-146a in the pathogenesis of CHBV.

## Subjects and methods

### Study subjects, and laboratory analyses

This case-control study included 100 patients who had confirmed CHBV infection, and 100 age and sex-matched controls with evidence of past HBV infection. All studied subjects were Egyptians. Patients were recruited from the outpatient clinic, Department of Internal Medicine, Faculty of Medicine, Fayoum University.

All study subjects had complete history taking; personal history, disease history (onset, course, and duration of the disease), medical history (other systemic disorders, and systemic treatment), physical examination, routine laboratory investigations as complete blood count, renal function tests, and liver function tests.

#### Cases

According to the Centers for disease prevention and control, a case of CHBV was identified by the absence of anti-HBc IgM (immunoglobulin M antibodies to HBV core antigen) and a positive result for any one of the following: HBsAg (hepatitis B surface antigen), HBeAg (hepatitis B e-antigen), or hepatitis B virus DNA (either quantitative, qualitative and genotype testing) [33].

In this study, the chronic infection was diagnosed with detectable immunoglobulin G (IGg) anti‐HBc and/or fibroscan (transient elastography), and any clinical findings compatible with chronic liver disease. Serum HBsAg, anti‐HBs, and anti‐HBc were assayed by enzyme-linked immunosorbent assay (ELISA) in the serum isolated from peripheral blood (Bioelisa, Biokit, Barcelona, Spain), according to the manufacturer’s instruction.

### Control subjects

Control subjects (CSs) who had past HBV infection (negative for HBsAg and positive for both anti-HBs and anti‐HBc), with normal liver function tests were collected in the same period. Those who had a history of HBV vaccination or were tested negative for anti-HBc and positive for anti-HBs were excluded from the study.

Informed written consent was collected from all the enrolled subjects at recruitment after a detailed, simplified explanation of the study. The study was approved by the research ethical committee, Faculty of Medicine, Fayoum University, and proceeded in agreement with the Helsinki declaration approved at the World Medical Association meeting in Edinburgh.

All the study subjects did not have a history of other liver diseases. The study subjects (patients or CSs) were excluded from the study in the following cases: 1) tested positive for anti-HIV or anti-HCV antibodies (Bioelisa, Biokit, Barcelona, Spain); 2) extreme alcohol consumption or smoking (smokes ≥1 cigarette pack(s)/daily, and 3) presence of other types of hepatic disorders, such as primary biliary cirrhosis, autoimmune hepatitis, hepatic carcinoma, non-alcoholic steatohepatitis, hemochromatosis, toxic hepatitis, Wilson’s disease, Budd-Chiari syndrome or α‐1 antitrypsin deficiency. None of the patients had previous history of immunosuppressant, or anti-viral treatment.

The sample size was calculated using (G power version 3). The minimal sample size of patients was 100 in each group needed to get power level 0.80, alpha level 0.05, and 15% as an expected difference in the proportion of subjects with TT in rs767649 between cases and control (25% vs.10%).

### Blood sample and Genomic DNA extraction

Venous blood samples were collected from 100 patients with CHBV infection, and 100 control subjects. One part of the sample was used for routine laboratory investigations and the other part was collected in EDTA tubes for DNA extraction from the whole blood and was stored at -80°C until further analysis. Genomic DNA was extracted from the peripheral blood samples using the QIAamp DNA Blood Mini kit (QIAamp; Qiagen, Hilden, Germany) following the manufacturer’s instructions. Quantitation of DNA was done utilizing the NanoDrop1-1000 spectrophotometer (NanoDrop innovations, Inc., Wilmington, USA). The HBV viral load (copies/mL) was assessed by real-time PCR (Rotor-gene Q Real-Time PCR system, Qiagen) using an Artus HBV RG qPCR Kit (Qiagen), which has a detection limit of 10 IU/mL, according to the manufacturer’s instructions.

### Genotyping

The miR-146a rs57095329 (A/G) and miR-155 rs767649 (T/A) polymorphisms were genotyped by real-time PCR (RT-PCR) using the customized TaqMan SNP Genotyping Assays; (C_2212229_10) for miR-155 rs767649 T>A and (C_90078480_10) miR-146a rs57095329 A>G (Applied Biosystems Thermo Fisher Scientific, Foster City, CA, USA) according to the manufacturer’s protocol.

The sequences of the primers used were: miR-155 rs767649 forward, 5’-ATATAACACATTATCAAAAACACTG-3’ and reverse, 5’-CACTTTTCTGAGTGCTCTAATCAGG-3’; and miR-146a rs57095329 forward 5’-CCCCGCGGGGCTGCGGAGAGTACAG-3’ and reverse, 5’-CAGGAAGCCTGGGGACCCAGCGCCT-3’.

Reactions were performed in a real-time PCR thermal cycler (Rotor-gene Q Real-Time PCR system, Qiagen). For amplification, reaction tubes were heated for 10 min at 95°C, followed by 45 cycles of 92°C for 15 s and 60°C for 90 sec.

Although real-time PCR is not the gold standard for genotyping polymorphisms; nowadays, it is the most commonly used technique in epidemiological studies involving a large number of subjects and frequencies of known polymorphisms. Genotyping quality control was performed in 10% of the samples, the amplification reactions were performed twice. By double-checking, a 100% rate of concordance between duplicates was found.

### In silico analysis of miR-155 rs767649 (T/A) polymorphisms and miR-146a rs57095329 (A/G)

These websites were used to analyze the miR-155 rs767649 (T/A) and miR-146a rs57095329 (A/G) SNPs:

#### Regulome DB

Is a database that marks up well-known and predicted SNPs and their relationship with regulatory factors in the intergenic regions of the genome. The reported regulatory DNA factors include the promoter regions that regulate transcription, transcription factors binding sites, and DNase hypersensitivity regions. The sources of data for the Regulome DB database were the published literature, public datasets from GEO, and the ENCODE project [34]. Regulome DB is available at https://regulome.stanford.edu/regulome-search/.

#### Haploreg v4.1

HaploReg is available at https://pubs.broadinstitute.org/mammals/haploreg/haploreg.php.

HaploReg website is a tool to read the non-coding genome at genotype block variants, such as SNPs located at disease-associated loci. It uses the linkage disequilibrium data from the 1000 Genomes Project. It can see protein binding sites, linked SNPs, chromatin state, small indels, SNPs effect on regulatory motifs, and SNPs effect on the expression from eQTL studies sequences conserved across mammals [35].

#### Alibaba 2.1

The putative transcription factor binding sites were detected within the empirically defined proximal promoters using AliBaba 2.1 (http://gene-regulation.com/pub/programs/alibaba2/index.html), which is based on the TRANSFAC 3.5 database [36].

### Statistical analysis

Divergence from Hardy-Weinberg equilibrium was tested for each polymorphism using Pearson’s Chi-square test by a specific calculator; available online at http://www.oege.org/software/hardy-weinberg.html. The collected data were statistically examined using SPSS (software statistical computer package) version 22 (SPSS Inc, USA). For quantitative data (clinical and laboratory characteristics), the mean and standard deviation (SD) were calculated. Independent-t-test or One way ANOVA was used in the comparison between any two groups or three groups, respectively. Qualitative data were presented as numbers and percentages, chi-square (χ2) was used as a test of significance. The Cochran-Armitage test for trend was used to evaluate the differences in the distribution of genotype between cases and controls. Adjusted Odds ratio (AOR) with 95% confidence intervals (CI) for the association of the study groups with different models and allelic frequency of the miR-146a A/G (rs57095329) and miR-155 T/A (rs767649) genotypes were calculated using univariate and multivariate logistic regression. For interpretation of the results of tests of significance, significance was adopted at *p* ≤ 0.05.

## Supporting information

S1 Table Predicted reference and altered sequence of miR-155 gene and miR-146a gene using Haploreg software. (DOCX)

S2 Table Predicted transcription factors binding sites affected by miR-155 rs767649 and miR-146a rs57095329 using Regulome DB, Haploreg and Alibaba software. (DOCX)

## Abbreviations

ALT: alanine aminotransferase
anti-HBcIgM: immunoglobulin M antibodies to HBV core antigen
AOR: Adjusted Odds ratio
CHBV: chronic HBV
CI: confidence intervals
CSs: Control subjects
ETS: E26 transforming sequence
Hb: hemoglobin
HBsAg: hepatitis B surface antigen
HBV: hepatitis B virus
IGg: immunoglobulin G
IKKβ: an inhibitor of nuclear factor kappa-B kinase subunit beta
IKKε: an inhibitor of nuclear factor kappa-B kinase subunit epsilon. IRF, interferon regulatory factor
MicroRNAs: MiRNAs
miR SNPs: SNPs in genes coding miRNAs
NF-αB: nuclear factor- alpha B
NF-κB: nuclear factor kappa B
PRDM1: PR domain zinc finger protein 1
SD: standard deviation
SNPs: Single nucleotide polymorphisms
TFs: transcription factors
