# Supplementary material for: miR-155 T/A (rs767649) and miR-146a A/G (rs57095329) single nucleotide polymorphisms as risk factors for chronic hepatitis B virus infection among Egyptian patients
Source: PLoS One. 2021 Aug 26;16(8):e0256724. doi: 10.1371/journal.pone.0256724 (PMC8389509; doi:10.1371/journal.pone.0256724)
Supplement: S1 Table — (DOCX) [file pone.0256724.s001.docx]

**Supplementary Table 1**: **Predicted reference and altered sequence of miR-155 gene and miR-146a gene using Haploreg Software.**

| **miR-155 rs767649** | **Reference Sequence:** | ATATATATAACACATTATCAAAAACACTG**T**CACTTTTCTGAGTGCTCTAATCAGGCAAT. |
| --- | --- | --- |
|  | **Altered Sequence:** | ATATATATAACACATTATCAAAAACACTG**A**CACTTTTCTGAGTGCTCTAATCAGGCAAT |
| **miR-146a rs57095329** | **Reference Sequence:** | ATTTCCCCGCGGGGCTGCGGAGAGTACAG**A**CAGGAAGCCTGGGGACCCAGCGCCTGACC |
|  | **Altered Sequence:** | ATTTCCCCGCGGGGCTGCGGAGAGTACAG**G**CAGGAAGCCTGGGGACCCAGCGCCTGACC |
