# Supplementary material for: miR-155 T/A (rs767649) and miR-146a A/G (rs57095329) single nucleotide polymorphisms as risk factors for chronic hepatitis B virus infection among Egyptian patients
Source: PLoS One. 2021 Aug 26;16(8):e0256724. doi: 10.1371/journal.pone.0256724 (PMC8389509; doi:10.1371/journal.pone.0256724)
Supplement: S2 Table — (DOCX) [file pone.0256724.s002.docx]

**Supplementary Table 2**: **Predicted transcription factors binding sites affected by miR-155 rs767649 and miR-146a rs57095329 using Regulome DB, Haploreg and Alibaba software.**

| **Software** | **Affected transcription factors binding sites** | |
| --- | --- | --- |
|  | **miR-155 rs767649** | **miR-146a rs57095329** |
| **Regulome DB** | IRF1, IRF2, PRDM1 | ELF3 |
| **Haploreg** | IRF, MRG1, HOXA9, PRDM1 | ETS, PAX-5, STAT |
| **Alibaba** | Presence of GCN4 in A allele (mutant allele) and absence of it in T allele (normal allele) | Found of ELK-1 in case of A allele (normal allele) which is replaced by MyoD and ETS-1 in case of G allele (mutant allele) |

IRF, the interferon regulatory factor; PRDM1, PR domain zinc finger protein 1; ETS, E26 transforming sequence; ELF3, E74 Like ETS Transcription Factor 3; MRG1, melanocyte-specific gene-related gene 1; HOXA9, Homeobox A9; **GCN4, General control protein GCN4;** PAX5, a member of the paired box (PAX) family of transcription factors; STAT, signal transducer and activator of transcription; ELK-1, ETS Like-1 protein; MyoD, myoblast determination protein 1[^[^](https://en.wikipedia.org/wiki/MyoD#cite_note-5)
